# Online interactive analysis of protein structure ensembles with Bio3D-web

**DOI:** 10.1093/bioinformatics/btw482

**Published:** 2016-07-16

**Authors:** Lars Skjærven, Shashank Jariwala, Xin-Qiu Yao, Barry J. Grant

**Affiliations:** ^1^Department of Biomedicine, University of Bergen, Bergen, Norway; ^2^Department of Computational Medicine and Bioinformatics, University of Michigan Medical School, Ann Arbor, MI, USA

## Abstract

**Summary:** Bio3D-web is an online application for analyzing the sequence, structure and conformational heterogeneity of protein families. Major functionality is provided for identifying protein structure sets for analysis, their alignment and refined structure superposition, sequence and structure conservation analysis, mapping and clustering of conformations and the quantitative comparison of their predicted structural dynamics.

**Availability:** Bio3D-web is based on the Bio3D and Shiny R packages. All major browsers are supported and full source code is available under a GPL2 license from http://thegrantlab.org/bio3d-web.

**Contact:** bjgrant@umich.edu or lars.skjarven@uib.no

## 1 Introduction

Many structures are now available for homologous proteins determined under different crystallization conditions and oligomerization states. Detailed comparison of these structures can inform on structural dynamic mechanisms critical for protein function including ligand binding, enzymatic catalysis, allosteric regulation and bi-molecular recognition. A wide range of bioinformatics tools and online servers enable researchers to explore and analyze individual biomolecular structures. Notable examples include molecular visualization, pairwise structural alignment and biophysics based tools including various normal mode analysis servers (Eyal *et al.*, 2015; Suhre and Sanejouand, 2004; Tiwari *et al.*, 2014). However, existing tools for detailed quantitative analysis of the sequence, structure and dynamics of large heterogeneous protein families often require significant computational expertise and typically remain accessible only to expert users with relevant programming skills. For example, the Bio3D package requires R (Grant *et al.*, 2006), ProDy requires python and Maven requires Matlab knowledge (Bakan *et al.*, 2011; Zimmermann *et al.*, 2011). A recent contribution to making such analysis more widely available online is the PDBFlex database (Hrabe *et al.*, 2016). PDBFlex catalogues the structural variation of the same protein (PDB structures sharing 95% or more sequence identity). However, more in-depth interactive analysis of user defined structure sets across species and diverse protein families is not currently available.

To this end we have developed Bio3D-web—a web application that implements a complete workflow for user customized investigation of protein sequence-structure-dynamic relationships. Bio3D-web provides unparalleled online functionality including inter-conformer relationship mapping with principal component analysis (PCA), and quantitative comparison of predicted internal dynamics across protein families via new ensemble normal mode analysis (eNMA). Together with conventional sequence and structure analysis methods these approaches allow researchers to map the structural dynamic properties of proteins for which PDB structures are available.

Bio3D-web requires no programming knowledge and thus decreases the entry barrier to performing advanced comparative sequence, structure and dynamics analysis. Bio3D-web is powered by the previously described Bio3D R package for structural bioinformatics (Grant *et al.*, 2006; Skjærven *et al.*, 2014). In particular, Bio3D approaches for identification of related protein structures, multiple alignment, rigid-core identification, optimal superposition, PCA and eNMA form the basis of the application (Supplementary Fig. S1). Structure and sequence annotations are derived from the RCSB PDB (Berman, 2000) and PFAM databases (Finn *et al.*, 2014). Bio3D-web employs Shiny’s reactive programming and web application framework to provide its interactive online interface (Shiny, http://shiny.rstudio.com).

## 2 Example application

Providing the PDB ID of a single Rho GTPase (1FTN) in the **SEARCH** tab identifies 1059 sequence similar structures from which the top 214 are automatically selected as the most related structures for further analysis. Users can optionally (de)select additional structures for further analysis. In this example we proceed with these structures that span RhoA/B/C, Cdc42 and Rac1/2/3 PDB entries. In the **ALIGN** tab the selected structures are subject to multiple alignment, similarity and conservation analysis. Characterization of superimposed structures is available from the **FIT** tab. This includes rigid core identification, conservation analysis and RMSD based clustering.

**Fig. 1.**
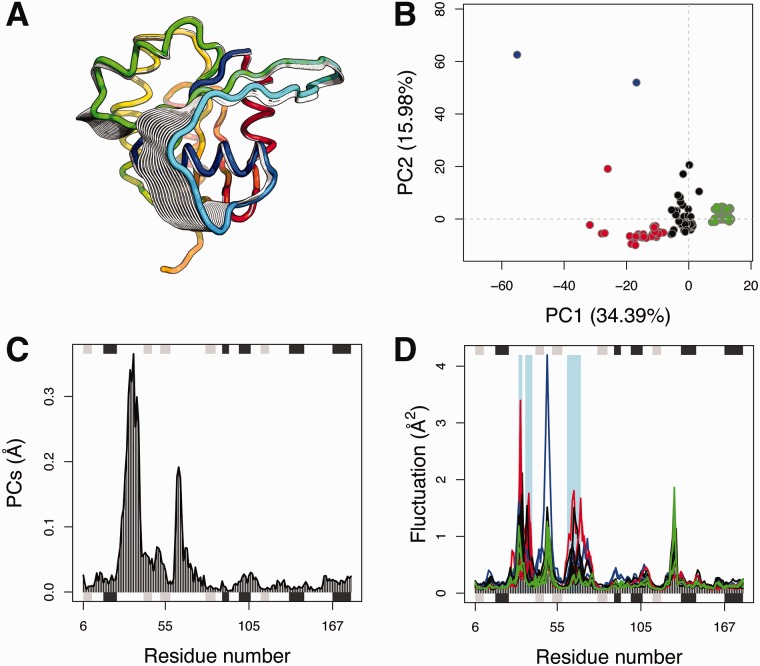
Bio3D-web analysis of Rho GTPases. (**A**) Visualization of the first principal component (PC) characterizing the major conformational variations. (**B**) Structures projected onto their two first PCs. Each point (or structure) is colored according to user specified criteria, in this case PCA-based clustering results. (**C**) Contribution of each residue to the first PC. (**D**) Ensemble normal mode analysis reveals the enhanced local dynamics of nucleotide free states (red) relative to the GTP- and GDP-bound (green and black) structures. Positions with significantly distinct flexibilities between states are indicated with light blue rectangles (*P* < 0.05) (Color version of this figure is available at *Bioinformatics* online.)

The **PCA** tab more clearly displays the relationship between all structures in terms of the principal displacements of their major variable regions (Fig. 1A). In particular, the interactive low-dimensional ‘conformer plot’, displaying structures projected onto user defined PCs, can be colored by sequence, RMSD and PCA based clustering results (Fig. 1B). This plot can also be clicked on to identify individual structures along with their annotations such as ligand bound species. For available Rho related structures three major conformational clusters are clearly apparent (black, green and red in Fig. 1B). Inspection of these structures in the linked annotation table reveals that the vast majority of cluster members correspond to GDP-bound, GTP-analogue bound and nucleotide free structures respectively. The residue-wise contributions to each PC are also displayed interactively highlighting a number of potentially key structural regions (Fig. 1C).

**eNMA** in the next tab indicates that these same regions display significantly distinct dynamics upon nucleotide exchange (Fig. 1D). Critically, the NMA here is performed on all selected structures in a way that facilitates the interpretation of their structural similarity and dissimilarity trends (Yao *et al.*, 2016). This allows a user to explore dynamic trends in all crystalized states in relation to each other without the conventional caveat of potentially over-interpreting the differences between extreme cases or a single artifactual structure. Collectively these results indicate the existence of three major Rho superfamily conformational states that differ by a collective displacement of two nucleotide-binding site regions, which in turn display significantly distinct flexibilities upon nucleotide binding. In the case of Rho GTPases these regions are known to be key for nucleotide dependent modulation of protein-protein interactions (Wittinghofer and Vetter, 2011). Further example application of Bio3D-web to a range of distinct protein families is presented as interactive demos online (http://thegrantlab.org/bio3d-web).

## 3 Conclusion

Bio3D-web is an online application for analyzing user defined heterogeneous biomolecular structure data. The design of Bio3D-web emphasizes simplicity over exhaustive inclusion of the many analysis methods available in the full Bio3D package. This effectively reduces the required technical expertise and thus facilitates advanced structural bioinformatics analysis for a broader range of researchers. In many cases it is envisaged that researchers will use Bio3D-web to understand general trends in their protein family of interest, which may then inform more specialized analyses. Bio3D-web is therefore designed to quickly explore biomolecular structure datasets and to act as a hypothesis-generating tool with sharable summary reports that capture all users defined analysis choices and optionally enable collaborators to visit previous analysis sessions.

## Supplementary Material

Supplementary Data
