# Supplementary material for: Online interactive analysis of protein structure ensembles with Bio3D-web
Source: Bioinformatics. 2016 Jul 16;32(22):3510–2. doi: 10.1093/bioinformatics/btw482 (PMC5181562; doi:10.1093/bioinformatics/btw482)
Supplement: Supplementary Data [file supp_32_22_3510__index.html]

Online interactive analysis of protein structure ensembles with Bio3D-web — Online interactive analysis of protein structure ensembles with Bio3D-web — Supplementary Data 

# Online interactive analysis of protein structure ensembles with Bio3D-web

## Supplementary Data

files

- Supplementary Data - pdf file
