# Supplementary material for: Online interactive analysis of protein structure ensembles with Bio3D-web
Source: Bioinformatics. 2016 Jul 16;32(22):3510–2. doi: 10.1093/bioinformatics/btw482 (PMC5181562; doi:10.1093/bioinformatics/btw482)
Supplement: Supplementary Data [file supp_btw482_Supplementary_information.pdf]

Supplementary information

Online interactive analysis of protein structure ensembles with Bio3D-web

Lars Skjærven[1], Shashank Jariwala[2], Xin-Qiu Yao[2], and Barry J. Grant[2,*]

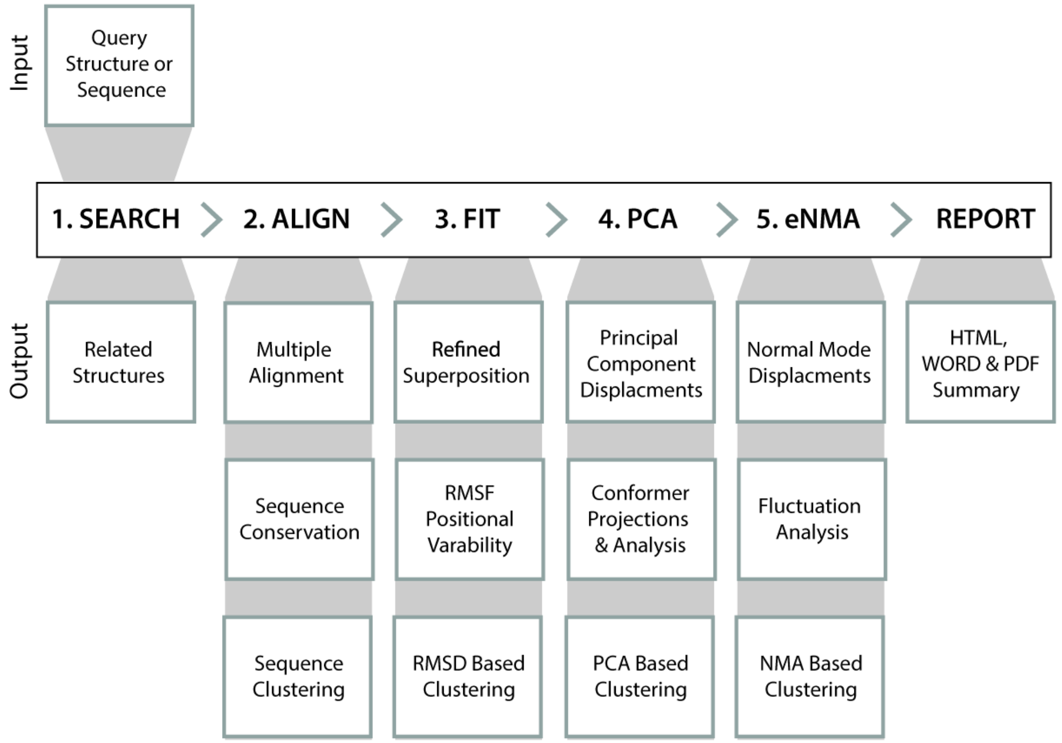

Figure S1: Bio3D-web overview. Bio3D-web takes a user provided protein structure or sequence as input in the SEARCH tab (**1**). The server provides a list of related structures, which can be selected for further analysis. (**2**) The ALIGN tab provides sequence alignment and analysis of the structures selected in the SEARCH tab. (**3**) In the FIT tab all structures are superimposed and visualized in 3D together with the results of conventional pair-wise structure analysis. (**4**) Principal component analysis of the structure set is performed in the PCA tab to characterize inter-conformer relationships. (**5**) Normal mode analysis on each structure can be carried out in the eNMA tab to explore dynamic trends for the available structural states.
